# Recognition and Awareness of Sepsis by First-Aid Providers in Adults With Suspected Infection: A Scoping Review

**DOI:** 10.7759/cureus.61612

**Published:** 2024-06-03

**Authors:** Amy Kule, Willem Stassen, Gustavo E Flores, Therese Djarv, Eunice Singletary

**Affiliations:** 1 Emergency Medicine, Loyola University Medical Center, Maywood, USA; 2 Emergency Medicine, University of Cape Town, Cape Town, ZAF; 3 Emergency Medicine, Emergency & Critical Care Trainings LLC, San Juan, PRI; 4 Emergency Medicine, Karolinska Institute, Stockholm, SWE; 5 Emergency Medicine, University of Virginia, Charlottesville, USA

**Keywords:** lay provider, first aid, identification, recognition, illness, infection, sepsis

## Abstract

Sepsis accounts for a significant proportion of preventable deaths worldwide and early treatment has been found to be a mainstay of decreasing mortality. Early identification of sepsis in the first-aid setting is critical as this results in a shorter time to hospital presentation and management with antibiotics and initial resuscitation. Our aim was to explore the existing literature related to either sepsis recognition or awareness of sepsis by first-aid providers who are evaluating an adult suspected of an acute infection.

Our scoping review was performed as part of the International Liaison Committee on Resuscitation's (ILCOR) continuous evidence evaluation process to update the 2024 ILCOR Consensus on Science with Treatment Recommendations. We searched Embase, Medline, and Cochrane databases from their inception to January 17, 2023, with updated searches performed on November 21, 2023, and December 2, 2023. The gray literature search was conducted on August 29, 2023. The population included adults presenting with an acute illness exhibiting signs and symptoms of a severe infection. Outcomes included sepsis recognition or awareness of sepsis by a lay first-aid provider.

After reviewing 4380 potential sources, four reviews (three systematic reviews and one scoping review), 11 observational studies, and 27 websites met the inclusion criteria. No study directly addressed our PICOST (Population, Intervention, Comparator, Outcomes, Study Design, and Timeframe) question as none were performed in the first-aid setting. Three systematic reviews and nine observational studies that assessed the ability of early warning scores to detect sepsis and predict adverse outcomes secondary to sepsis had inconsistent results, but many found the screening tools to be useful. One scoping review and one observational study found public knowledge and awareness of sepsis to be variable and dependent upon healthcare employment, location, education level, ethnicity, sex, and age. Signs and symptoms associated with sepsis as listed by gray literature sources fell primarily under nine general categories as a means of educating the public on sepsis recognition.

Although this scoping review did not identify any studies that directly addressed our outcomes, it highlights the need for future research to better understand the recognition of sepsis in first-aid settings.

## Introduction and background

Sepsis is a critical medical condition characterized by a dysregulated host response to infection, leading to organ dysfunction and potential mortality [1]. With its potential for rapid progression and poor outcomes, frequently requiring admission to the medical wards or an intensive care unit (ICU), sepsis poses a significant burden to healthcare systems worldwide [2]. While the precise global incidence of sepsis is difficult to ascertain, estimates suggest that millions of cases occur annually and it is a major public health issue [3]. Hospital mortality rates have been reported as ranging between 15% and 30% in high-income countries (HICs), increasing to 50% or more in low- to middle-income countries (LMICs) [4,5].

Sepsis presents with a diverse array of signs and symptoms that can vary depending on the underlying infection, the patient's age, and comorbidities. Common clinical manifestations include fever, lethargy, tachycardia, tachypnea, altered mental status, and subjectively feeling unwell, although these may or may not be present. The non-specific nature of these features often results in sepsis mimicking other conditions and diagnostic uncertainty [6].

Prompt recognition and early intervention are paramount in managing sepsis effectively. Early administration of appropriate antibiotics and resuscitative measures have been shown to improve outcomes, including reducing mortality rates [7-9]. Identifying sepsis in its early stages has proven to be challenging, even for healthcare providers with access to advanced testing. Screening tools have been developed to assist with the identification of sepsis, examples of which include vital signs, Systemic Inflammatory Response Syndrome (SIRS) criteria, quick Sequential Organ Failure Score (qSOFA) criteria, or Sequential Organ Failure Assessment (SOFA) criteria, National Early Warning Score (NEWS), NEWS2, Modified Early Warning Score (MEWS), and Phoenix Sepsis Score [10-13]. However, the utility of these scores has been limited to the healthcare setting. A major limitation to these tools is that they cannot be universally applied by those assessing an acutely ill person for possible sepsis, specifically the lay provider who is often the initial contact and lacks the ability to obtain some of the required variables. Consequently, first-aid providers are limited to more basic means of evaluating someone suspected of sepsis, relying solely on subjective and objective signs of infection and hypoperfusion.

Despite the critical importance of early recognition and intervention, there is limited research evaluating a first-aid provider’s ability to identify sepsis. We sought to explore the existing literature that was related to either sepsis recognition or awareness of sepsis by first-aid providers who are evaluating an adult suspected of an acute infection.

## Review

This scoping review was developed as part of the International Liaison Committee on Resuscitation's (ILCOR) continuous evidence evaluation process, conducted by the ILCOR First Aid Task Force [14] and was reported according to the Preferred Reporting Items for Systematic Reviews and Meta-Analyses extension for Scoping Reviews (PRISMA-ScR) [15].

Questions and objectives

We sought to answer the following PICOST (Population, Intervention, Comparator, Outcome, Study Designs and Timeframe) question, which was defined as:

Population: Adults who are being evaluated by a first-aid provider for an acute illness.

Intervention: The identification of any specific signs or symptoms (e.g., pale, blue, or mottled skin, lips or tongue, gums, nails; non-blanching rash; difficulty in breathing or rapid respiratory rates; rigors/shivering; lack of urination in a day; muscle pain; confusion or slurred speech).

Comparator: Fever (≥ 38°C) with signs of infection.

Outcome: 1. Recognition of a seriously ill person requiring hospitalization or evaluation by a physician for sepsis, 2. Increased awareness of sepsis.

Study designs: Randomized controlled trials (RCTs) and non-randomized studies (non-randomized controlled trials, interrupted time series, controlled before-and-after studies, cohort studies) are eligible for inclusion. Gray literature and social media and non-peer reviewed studies, unpublished studies, conference abstracts, and trial protocols are eligible for inclusion. All relevant publications in any language are included as long as there is an English abstract.

Timeframe: All years.

Inclusion and exclusion criteria

Our population included adults (aged 18 years and older) experiencing an acute illness exhibiting signs and symptoms concerning sepsis as suspected in the prehospital setting. We excluded opinions and consensus papers.

Information sources and search strategies

We conducted our search using three separate strategies: (1) published literature through Embase, Medline, and Cochrane databases; (2) hand-searching for relevant articles; and (3) gray literature search using Google.com in an attempt to identify knowledge gaps in this area.

Peer-Reviewed Literature

With the assistance of an information specialist, an initial search strategy was developed and executed in Embase, Medline, and Cochrane Database of Systematic Reviews and Cochrane Central Register of Controlled Trials on January 17, 2023 (Appendix 1). Updated searches were performed on November 21, 2023, and again on December 2, 2023, to comply with protocols outlined by the ILCOR leadership (Appendixes 2, 3). 

Gray Literature

In order to supplement the database search and identify the current initiatives geared toward educating the public on sepsis recognition, gray literature searches were performed on August 29, 2023, through Google.com using key terms “Sepsis” and “First Aid” (747,000 results); “Sepsis Recognition” and “Public” (15,900 results); and “Sepsis Recognition” and “Signs and Symptoms” (38,300,000 results) (Appendix 4). The following were criteria for inclusion: (1) primary website from a sepsis awareness campaign, healthcare organization, first-aid training course, or government sepsis program initiative; (2) listed specific signs and symptoms for sepsis identification. The results were excluded if healthcare providers were the targeted audience.

Screening and selection of sources

Peer-Reviewed Literature

**Figure 1:**
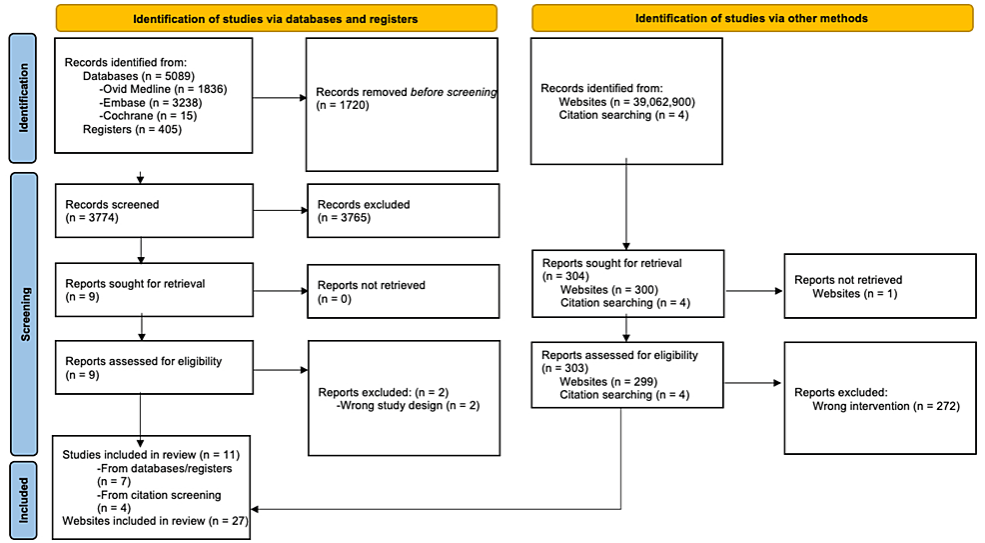
PRISMA Diagram of Included Studies PRISMA: Preferred Reporting Items for Systematic Reviews and Meta-Analyses

The initial search retrieved 5494 results, and after removing duplicates, 3774 unique articles remained for title and abstract screening. Records were downloaded and imported into an EndNote (version 21, Clarivate, Philadelphia, PA, 2013) database to facilitate removal of duplicates and screening. Four independent reviewers (AK, WS, TD, and GF) screened titles and abstracts using Rayyan [16] (www.rayyan.ai), a web-based tool, after which nine articles were identified for full-text review. Four additional articles were identified from a handpicked search. Discrepancies between the reviewers were resolved through discussion after which eleven studies met the final inclusion criteria. A summary of the screening process is shown in the PRISMA diagram (Figure 1). Updated searches performed on November 21, 2023, and December 2, 2023, retrieved 302 results available for screening. Nine articles underwent full-text review, after which four were included.

Gray Literature

One reviewer (AK) screened the first 100 titles of each search for relevance, and after removal for redundancy, a total of 27 were selected for inclusion in the review.

Data extraction and analysis

Data from included data sources were extracted into an extraction matrix that was developed a priori. Data were subjected to simple descriptive analysis and organized into major topic domains and presented narratively. As is permitted in a scoping review, no formal risk of bias assessments were undertaken.

Results

Study Characteristics

We identified three systematic reviews [17-19], one scoping review [20], and 11 observational studies [21-31] for inclusion, all of which were conducted in HICs; none were performed in the first-aid setting. No published studies directly addressed a first-aid provider’s recognition of sepsis through the presence of specific signs or symptoms in a seriously ill person with the subsequent need to seek further medical attention. Given the lack of any direct evidence, we included studies that were performed in either the prehospital or in-hospital settings, or by emergency medical service (EMS) providers, and extrapolated the data to suggest relevance to the first-aid setting. Studies evaluating physiologic variables that a lay provider could obtain in a first-aid setting, such as temperature, heart rate, and respiratory rate, either in isolation or when assessing using clinical scoring tools, were selected for inclusion. Seven studies assessed screening score performance in the prehospital or in-hospital setting to identify sepsis [17-19,22-25]. One study explored the clinical signs and field assessments by emergency medical service providers of patients with and without a suspicion of sepsis [29]. Five studies evaluated prehospital and emergency triage screening score prediction for adverse in-hospital outcomes (ICU admission or mortality) [21,26,28,30,31]. Two studies evaluated the awareness and knowledge of sepsis by various members of the population and identified potential contributing factors affecting the results [20,27]. The characteristics of the included studies are provided in Tables 1, 2.

**Table 1:** Characteristics of and Findings in the Included Review Studies Abbreviations: ED, emergency department; EMS, Emergency Medical Services; HR, heart rate; qSOFA, quick Sequential Organ Failure Assessment; SIRS, Systemic Inflammatory Response Syndrome; RR, respiratory rate

Author, year	Study design	Number of articles identified (n)	Population	Intervention	Key findings	Conclusions
De Silva et al. 2023 [17]	Systematic review	(n=18)	People with suspected or confirmed sepsis in the emergency setting	Compared sensitivity and specificity of qSOFA to 31 other screening tools for the diagnosis of sepsis	qSOFA produced high specificity and low sensitivity for emergency diagnosis of sepsis among the included studies.	No single screening tool is recommended at this time for use in the emergency setting. A combination of qSOFA and SIRS can improve the prognostic accuracy of 30-day mortality for ED presentation.
Lane et al. 2016 [18]	Systematic review	Total: (n=16); Identification: (n=8); Management: (n=7); Both: (n=1)	Articles describing the accuracy of prehospital sepsis identification and prehospital management of patients with sepsis, severe sepsis, or septic shock by EMS	None	The most common approach to identification involved applying SIRS criteria or a combination of vital signs. Primary vital signs considered in seven studies: Temperature, HR, RR.	EMS providers can identify patients with sepsis with modest sensitivity and specificity. The use of provider impression alone had poor sensitivity. Fluid resuscitation was the most commonly described treatment.
Smyth et al., 2016 [19]	Systematic review	Total: (n=9); Development of tool: (n=3); Paramedic diagnosis of sepsis: (n=6)	Articles addressing the identification of sepsis among adult patients managed prehospital by EMS. Studies either addressed paramedic diagnosis of sepsis or the development of prehospital sepsis screening tools	None	Very low quality of evidence addressing recognition of sepsis by EMS using a screening tool. The majority of the screening tools rely on SIRS criteria. Very low quality of evidence addressing the accuracy of EMS diagnosis of sepsis in clinical practice.	The accuracy of sepsis recognition by EMS is variable. Prehospital screening tools need to be validated in clinical practice.
Fiest et al. 2022 [20]	Scoping review	Total: (n=80); Patients: (n=13); Public: (n=15); Healthcare professionals: (n=48)	Articles related to sepsis awareness, knowledge, and information-seeking behaviors among patients, public, and healthcare professionals	None	Awareness and knowledge of sepsis is high among healthcare professionals compared to patients/public. Patient/public awareness of sepsis gradually improved over time. Most patients/public get sepsis information from the Internet.	Awareness and knowledge of sepsis among patients, public, and healthcare professionals varied globally.

 

**Table 2:** Characteristics of and Findings in the Included Observational Studies Abbreviations: qSOFA, Quick Sequential Organ Failure Assessment; SIRS, Systemic Inflammatory Response Syndrome; ED, emergency department; EMS, emergency medical services; HR, heart rate; RR, respiratory rate; SI, Shock Index; ICU, intensive care unit; OR, odds ratio; CI, confidence interval; PPV, positive predictive value; NEWS2, National Early Warning Score 2; mSOFA, Modified Sequential Organ Failure Assessment; AUC, area under the receiver operating characteristic curve; PRESEP, prehospital early sepsis detection; AMS, altered mental status; SD, standard deviation; tqSOFA, Triage Quick Sequential Organ Failure Assessment; CAP, community-acquired pneumonia; CCS, critical care support; LR, likelihood ratio; MEWS, Modified Early Warning Score PROGRESS: Place of residence, Race/ethnicity/culture/language, Occupation, Gender/sex, Religion, Education, Socioeconomic status, Social capital Plus: 1. Personal characteristics associated with discrimination (e.g. age, disability) 2. Features of relationships (e.g. smoking parents, excluded from school 3. Time-dependent relationships (e.g. leaving the hospital, respite care, other instances where a person may be temporarily at a disadvantage) CURB-65: confusion, uremia, respiratory rate, blood pressure, age>65 years

Author, year	Type of study	Location, study size (n)	Population	PROGRESS-Plus characteristics	Intervention	Comparison	Outcomes	Key findings	Conclusions
Baez et al. 2013 [21]	Cross-sectional descriptive study	United States of America (n=63)	Adults (≥18 years) transported by EMS with the diagnosis of sepsis	N/A	EMS data, including vital signs (temperature, HR, RR, SI, and association to outcomes	None	Primary: 1. Mortality, 2. Admission to ICU; Secondary: 1. Ventilator days, 2. ICU length of stay, 3. Hospital length of stay	Elevated RR was found to predict ICU admissions (OR 4.81 (CI, 1.16-21.01; P = .0116)). No physiologic variables studied were found to predict mortality.	Out-of-hospital shock index and respiratory rate have high predictability for ICU admission.
Barbara et al. 2018 [22]	Retrospective chart review	United States of America (n=72)	Adults (≥18 years) transported by EMS and evaluated in ED	1. Gender (No observed discrepancy relating to gender)	Identification of EMS patients meeting all three qSOFA criteria	None	ED identification of sepsis ((≥2 SIRS criteria or admitting diagnosis of sepsis)	Prehospital qSOFA PPV of 66.7% (95% CI 55.8-77.6).	EMS patients with positive qSOFA more likely to be diagnosed with sepsis in the ED.
Goodacre et al. 2023 [23]	Retrospective diagnostic cohort	United Kingdom (n=12,870)	Adults (≥18 years) transported by EMS to the ED	1. Sex; 2. Ethnicity, 3. Excluded mental health problems	Accuracy of prehospital early warning scores + paramedic diagnostic impression for identifying sepsis.	None	Identification of sepsis requiring urgent treatment	No combination of early warning score + paramedic diagnostic impression provided greater sensitivity than 0.8 and PPV greater than 0.15 for sepsis. NEWS2 + paramedic diagnostic impression of infection or sepsis identified 1/3 to 1/2 of sepsis cases.	NEWS2 had superior accuracy over all other early warning scores.
Melero-Guijarro et al. 2023 [24]	Prospective, multicenter cohort study	Spain (n=535)	Adults (≥18 years) with suspected infection transferred by EMS to the ED	1. Age, 2. Sex, 3. Nursing home residence	Performance of qSOFA, NEWS2, and mSOFA to identify sepsis in prehospital care	None	Primary: In-hospital sepsis diagnosis; Secondary: 1. diagnosis of septic shock, 2. Two-day in-hospital mortality	No statistically significant differences were found for sepsis or septic shock. Only the comparison between scores for mortality outcome presented statistically significant differences. mSOFA outperformed the other two scores (p < 0.001 vs. both scores) in identifying two-day mortality.	The mSOFA score performed consistently better in two-day mortality prediction and diagnosis of septic-shock than NEWS2 and qSOFA.
Nualprasert et al. 2024 [25]	Retrospective observational study	Thailand (n=354)	Adults (≥18 years) transported by EMS to the ED	1. Sex, 2. Age	The ability of PRESEP score and Miami Sepsis Score to predict sepsis in the prehospital setting	None	Final diagnosis of sepsis within 48 hours of admission	AUC for the PRESEP score was 0.83 (95% confidence interval [CI] 0.79–0.88) AUC for Miami Sepsis Score was 0.80 (0.75–0.85). The sepsis group compared to those without sepsis showed a high proportion of patients with worse vital signs.	Due to their relatively high ability to detect sepsis patients, the PRESEP and Miami Sepsis Scores are useful tools to screen septic patients in prehospital settings.
Olander et al. 2019 [26]	Retrospective observational study	Sweden (n=327)	Adults (≥18 years) transferred by EMS to the ED and started on antibiotic treatment within 48 hours for suspected sepsis	1. Gender, 2. Age, 3. Form of housing, 4. Type of housing assistance	Prehospital characteristics of patients with sepsis while transported by EMS	None	Patients with and without adverse in-hospital outcomes (ICU requirement or in -hospital mortality).	50 patients had adverse outcomes. Prehospital AMS, decreased body temperature, decreased oxygen saturation, and increased serum glucose were associated with an adverse outcome.	Altered mental status, low temperature, low oxygen saturation, and high serum glucose level may be early prehospital characteristics related to poorer prognosis and adverse outcomes in patients with sepsis.
Parsons Leigh et al. 2022 [27]	Cross-sectional survey	Canada (n=3200)	Representative sample of English- and French-literature adults (≥18 years) who resided in Canada	1. Sex, 2. Age, 3. Education, 4. Annual household income, 5. Ethnicity, 6. Region of residence	Examined public awareness and knowledge of sepsis	None	1. Awareness of sepsis, 2. Knowledge of sepsis, 3. Sepsis information access	Overall 61.4% heard of sepsis. There was significant regional variation in self-reported awareness (p<0.001) and significant association with respondents’ education, ethnicity, sex, and age. Relatively few (9.5%) respondents perceived their level of knowledge as good (7.7%) or very good (1.8%). Respondents who had heard of sepsis scored an average of 44% (mean=44.3% SD 18.9%) on knowledge-based questions. The strongest predictors of sepsis knowledge were previous exposure to sepsis, healthcare employment, female sex, and a college/university education (p < 0.001, all). The top recognized descriptor of sepsis was “the body’s extreme response to infection” (61.3%) and the top recognized sign/symptom of sepsis was fever (55.7%). One-fifth (19.7%) of the respondents who had heard of sepsis (61.8%) actively looked for information and a majority (68.5%) used the Internet.	We found an incomplete awareness and understanding of sepsis among adults.
Perman et al. 2020 [28]	Retrospective observational cohort study	United States of America (n=2859)	Adults (≥18 years) transported by EMS, admitted from the ED with severe sepsis present on admission, with treatment initiated in the ED	1. Age, 2. Gender, 3. Race	Assessed triage qSOFA, maximum qSOFA (derived from worst vitals during ED stay), and first initial serum lactate	Triage SIRS criteria	In-hospital mortality	Mortality for tqSOFA<2 was 11.7% and for tqSOFA≥2 mortality was 26.4%. Mortality for patients with maximum qSOFA<2 was 8.5% and for maximum qSOFA≥2 was 20.6%. Sensitivity of tqSOFA≥ 2 and maximum qSOFA≥ 2 to predict in-hospital mortality were 33% and 69%, respectively. Triage SIRS criteria and the initial lactate > 3 mmol/L had sensitivities of 82% and 65%, respectively.	Triage qSOFA performed poorly at identifying patients at an increased risk of mortality on initial presentation. qSOFA cannot be relied upon to identify sepsis patients at high risk of death.
Sjösten et al. 2019 [29]	Retrospective observational study	Sweden (n=353)	Patients transported by EMS and admitted with a final diagnosis related to sepsis	1. Age, 2. Gender	EMS field assessments of patients with prehospital suspicion of sepsis	EMS field assessments of patients without prehospital suspicion of sepsis	Hospital discharge diagnosis codes corresponding to sepsis	Sepsis symptoms included in research protocol: abdominal pain, dyspnea, diarrhea, nausea/vomiting, rigors/shivering, skin rash/hives, and confusion. Dyspnea, rigors/shivering, and confusion were the most common symptoms, and higher respiratory and heart rate, and temperature abnormalities were most common abnormal vital signs noted in patients with a prehospital suspicion of sepsis.	EMS clinicians identified 36% of patients in the prehospital phase with a final hospital diagnosis of sepsis.
Spagnolello et al. 2021 [30]	Retrospective observational study	Italy (n=505)	Adults (≥18 years) evaluated in a single ED with an ED diagnosis of CAP	1. Age, 2. Gender	Assessed the ability of qSOFA and CURB-65 to predict mortality in the ED	None	1. Mortality in the ED, 2. CCS requirement, 3. ICU admission	Positive qSOFA (≥ 2) had a LR of 11 for mortality compared to qSOFA negative (0-1) patients in CAP patients.	qSOFA is a valuable score for predicting mortality in the ED and for the prompt identification of patients with CAP requiring CCS.
Usul et al. 2021 [31]	Retrospective cohort study	Turkey (n=266)	Adults (≥18 years) transported by EMS, admitted to the hospital with a diagnosis of sepsis	1. Age, 2. Gender	Assessed qSOFA and MEWS to predict ICU hospitalization and 28-day mortality	None	1. 28-day mortality, 2. ICU admission	MEWS value > 4 (AUC = 0.641; sensitivity 76.98; specificity 44.88; +LR: 1.4, −LR:0.51; p < 0.001; 95% CI: 0.575–0.708) and qSOFA score > 1 (AUC = 0.651; sensitivity 84.89; specificity 37.8; +LR: 1.36, −LR: 0.4; p < 0.001; 95% CI: 0.585–0.717) were both significant in predicting mortality. MEWS value >5 and qSOFA score >1 were both significant in predicting ICU hospitalization.	MEWS and qSOFA can be used to predict ICU hospitalization and mortality in patients diagnosed with sepsis.

Screening Score Performance to Identify Sepsis 

Three systematic reviews [17-19] and four observational studies [22-25] evaluated the performance of sepsis screening scores to identify adult patients with sepsis in both the prehospital and in-hospital settings and found the accuracy of sepsis recognition by EMS providers to be variable. De Silva et al. identified 18 studies that compared the sensitivity and specificity of qSOFA to 31 other screening tools for the diagnosis of sepsis and determined that qSOFA produced high specificity and low sensitivity for the emergency diagnosis of sepsis among people with suspected or confirmed sepsis in either the prehospital setting or Emergency Department (ED) [17]. Lane et al. described in a narrative review of 17 articles that the accuracy of prehospital sepsis identification of septic patients by EMS using SIRS criteria or a combination of vital signs was heterogeneous [18]. The most common vital signs taken into consideration in seven studies were temperature, heart rate, and respiratory rate. Smyth et al. discussed very low quality of evidence in six studies that addressed the recognition of sepsis by EMS with the majority of screening tools relying on SIRS criteria [19].

One prospective, ambulance-based, cohort study analyzed the performance of qSOFA, NEWS2, and modified SOFA (mSOFA) as sepsis predictors in the prehospital setting among patients with a suspected infection and did not find any statistically significant differences between the scores [24]. Barbara et al. performed a retrospective chart review of patients that met all the three qSOFA criteria by EMS and found a sepsis diagnosis in the ED to have a positive predictive value of 66.67% (95% CI 55.8-77.6) [22]. The ED septic patients demonstrated a statistically significant difference of higher average temperature by >2 °F (>1 °C) (p=0.001), mean maximum heart rate by a difference of >10 beats per minute (p=0.019), and mean maximum respiratory rate of >4 breaths per minute (p=0.0001) compared with the non-septic patients. A retrospective diagnostic cohort study evaluated the accuracy of 21 prehospital early warning scores in combination with parametric diagnostic impression for identifying sepsis and found NEWS2 to be superior to all other scores [23]. A retrospective analysis by Nualprasert et al. evaluated the ability of two early warning scores, Prehospital Early Sepsis Detection (PRESEP) score and Miami Sepsis Score, to detect septic patients in the prehospital setting and found both to be potential screening tool options for EMS given the relatively high sensitivities at the given cutoff values, 0.83 (0.73-0.90) and 0.81 (0.71-0.89), respectively [25].

EMS Use of Clinical Signs and Assessments in Suspected Sepsis

Sjösten et al. performed a retrospective observational analysis of EMS field assessments of patients with a prehospital suspicion of sepsis during which symptoms and vital signs were recorded in the research protocol [29]. Prehospital signs and symptoms that were identified in patients with sepsis included: respiratory difficulties, gastrointestinal symptoms, altered mental status, skin rash, rigors or shivering, temperature abnormalities, and tachycardia. The most common symptoms were dyspnea, rigors or shivering, and confusion, and elevated respiratory and heart rate as well as temperature abnormalities were the most common abnormal vital signs observed in patients with a prehospital suspicion of sepsis.

Prediction of Adverse Outcomes

One systematic review [17] and four observational studies [24,28,30,31] evaluated the prognostic ability of sepsis screening tests performed in the prehospital or emergency setting to predict adverse in-hospital outcomes, such as progression to septic shock, mortality, critical care intervention, or ICU requirement. The qSOFA score was universally evaluated in all the studies but with variable results.

Based on the 18 articles included in the review conducted by De Silva et al., the authors concluded that qSOFA most successfully predicted mortality in at-risk patients compared to 31 other screening tools [17]. A retrospective observational study that evaluated patients presenting to the ED with community-acquired pneumonia also found the qSOFA score to be useful in predicting ED mortality and identifying septic patients requiring critical care support [30]. Usul et al. similarly concluded that a qSOFA score >1 (AUC = 0.651; sensitivity 84.89; specificity 37.8; +LR: 1.36, -LR: 0.4; p<0.001; 95% CI: 0.585-0.717) and MEWS score >4-5 (AUC = 0.641; sensitivity 76.98; specificity 44.88; +LR: 1.4, -LR: 0.51; p<0.001; 95% CI: 0.575-0.708) could be utilized to predict mortality and ICU hospitalization in patients diagnosed with sepsis [31].

A comparison of qSOFA, NEWS2, and mSOFA in a prospective, multicenter cohort study showed that the mSOFA score performed consistently better in two-day mortality prediction and diagnosis of septic shock than both NEWS2 and qSOFA [24]. However, the unique variables of mSOFA differentiating it from NEWS2 and qSOFA are derived from blood work. Perman et al. calculated qSOFA scores obtained at triage and during the ED stay derived from the worst vital signs to predict in-hospital mortality and compared these results with triage SIRS criteria of patients admitted with severe sepsis [28]. Sensitivities for the prediction of in-hospital mortality of triage qSOFA, maximum qSOFA, and triage SIRS were 33%, 69%, and 82%, respectively, suggesting that triage qSOFA is not reliable at identifying septic patients at a high risk of death.

Two observational studies [21,26] assessed prehospital characteristics of patients who were admitted to the hospital with diagnosed or suspected sepsis for association with adverse outcomes. One retrospective, cross-sectional descriptive study found that rapid respiratory rate had high predictability for ICU admission (OR 4.81 (CI, 1.16-21.01; P = .0116)) but no physiologic variables were predictive of mortality [21]. Among the 327 patients who were admitted for suspected sepsis and initiation of treatment with antibiotics in a retrospective observational study by Olander et al., it was noted that the presence of prehospital altered mental status and low temperature may be related to poorer prognosis and adverse outcomes with need for ICU level of care and mortality [26].

Awareness and Knowledge of Sepsis

A review of 80 articles related to sepsis awareness and knowledge among healthcare professionals, patients. and the general public found that both knowledge and awareness varied significantly across the groups and geographically [20]. Patients and the public, most of whom obtained information from the Internet, had less awareness and knowledge of sepsis than healthcare professionals. One cross-sectional survey evaluating public awareness and knowledge of sepsis in Canada found significant regional variation in self-reported awareness (p<0.001) and significant association with the respondents’ education, ethnicity, sex, and age [27]. The most recognized sign or symptom of sepsis was “fever” (55.7%) and others being “infection” (52.9%), “feeling extremely ill (like you are going to die)” (39.3%), “extreme shivering or muscle pain” (27.6%), “fast heart rate” (26.4%), “fast breathing/severe breathlessness” (21.2%), “skin blotchy or discolored” (20.7%), “slurred speech or confusion” (12.5%), and “passing no urine all day” (8.0%).

Gray Literature

Of the 27 sources [32-58] that were included from the gray literature, 13 [32-44] were organizations that focused on first-aid training, one [45] was a government-based sepsis public awareness campaign workgroup, and 13 [46-58] were sepsis-focused campaign initiatives, major healthcare institutions, or government-run sepsis- or disease-focused organizations. Each source listed sepsis-associated signs and symptoms as a guide to the public on sepsis recognition as outlined in Table 3.

**Table 3:** Characteristics of Included Gray Literature Sources

Search #	Organization	Website Title	Website	Temperature	Neurology	Musculoskeletal	Urinary	Respiratory	Skin	Subjective	Cardiac	Gastrointestinal	Infectious Disease
Search #1 - Sepsis and “First Aid”	St John Ambulance [32]	Sepsis in Adults and Older Children	https://www.sja.org.uk/get-advice/first-aid-advice/sepsis/sepsis-in-adults-and-older-children/		Slurred speech, confusion, dizziness, or faintness	Extreme shivering or muscle pain	Passing no urine over the past 24 hours	Severe breathlessness or rapid breathing	Skin is mottled, pale, or discolored	"I feel sicker than I ever have before"			
Search #1 - Sepsis and “First Aid”	Scottish Ambulance Service [33]	Identifying Sepsis	https://www.scottishambulance.com/first-aid-education/identifying-sepsis/	Very high or low temperature	Confusion	Uncontrolled shivering	Not passing as much urine		Cold or blotchy hands				
Search #1 - Sepsis and “First Aid”	First Aid for Life [34]	Sepsis – What to Look Out for	https://firstaidforlife.org.uk/sepsis/		Slurred speech	Muscle pain and shivering	Failure to pass any urine	Breathlessness	Pale, mottled skin	A sense of ‘impending doom’ or a feeling that they might die			
Search #1 - Sepsis and “First Aid”	CPR First Aid [35]	What Is Septic Shock and How to Prevent It	https://cprfirstaid.com.au/what-is-septic-shock-and-how-to-prevent-it/	Fever and chills	Confusion or disorientation			Shortness of breath	Sweaty or clammy palms and skin		Elevated heart rate		
Search #1 - Sepsis and “First Aid”	Andersson First Aid Training [36]	What Is Sepsis?	https://anderssonfirstaidtraining.co.uk/blog/what-is-sepsis/		Slurred speech or confusion	Extreme shivering or muscle pain	Passing no urine (in a day)	Severe breathlessness	Skin mottled or discolored	It feels like you’re going to die			
Search #1 - Sepsis and “First Aid”	ANZCOR [37]	Recognition and First Aid Management of the Seriously Ill Person Including Sepsis	https://www.anzcor.org/assets/anzcor-guidelines/guideline-9-2-12-recognition-and-first-aid-management-of-the-seriously-ill-person-including-sepsis-273.pdf	Fever or feeling very cold	Restlessness, agitation, dizziness, decreased level of consciousness, confusion, slurred speech, or disorientation	Shivering or shaking; unexplained muscle pain or discomfort	Passing little or no urine	Rapid breathing; breathlessness or feeling short of breath	New rash or blotchy, pale, or discolored (often described as mottled) skin	The person may say they "don’t feel right" or they might say they feel like they "are going to die"	Rapid heart rate	Nausea and or vomiting	
Search #1 - Sepsis and “First Aid”	HTS Training [38]	6 Clues to Spotting Sepsis	https://hts-training.co.uk/sepsis-symptoms/		Slurred speech or confusion	Extreme shivering or muscle pain	Passing no urine (in a day)	Severe breathlessness	Skin mottled or discolored	I feel like I might die			
Search #1 - Sepsis and “First Aid”	SkillBase First Aid [39]	Could It Be Sepsis?	https://www.skillbasefirstaid.com/could-it-be-sepsis/	A high temperature (fever) or low body temperature	Feeling dizzy or faint; A change in mental state – such as confusion or disorientation; slurred speech; loss of consciousness	Severe muscle pain	Less urine production than normal – for example, not urinating for a day	Severe breathlessness	Cold, clammy, and pale or mottled skin		A fast heartbeat	Diarrhoea; nausea and vomiting	
Search #1 - Sepsis and “First Aid”	First Response [40]	Sepsis Awareness - First Response	https://www.firstresponse.org.uk/medical-training/sepsis	A high temperature (fever) or low body temperature	Feeling dizzy or faint; a change in mental state – such as confusion or disorientation; slurred speech; loss of consciousness	Severe muscle pain	Less urine production than normal – for example, not urinating for a day	Severe breathlessness	Cold, clammy, and pale or mottled skin		A fast heartbeat	Diarrhoea, nausea, and vomiting	
Search #1 - Sepsis and “First Aid”	The Hippocratic Post [41]	Sepsis Signs and Symptoms	https://www.hippocraticpost.com/first-aid/sepsis-signs-and-symptoms/	Fever	Dislike bright lights; drowsy, difficult to wake; convulsions/seizures; severe headache; confusion and irritability	Stiff neck; severe muscle pain			Pale, blotchy skin; spots/rash; cold hands and feet			Vomiting	
Search #1 - Sepsis and “First Aid”	NR Medical Training [42]	Understanding Sepsis: Early Detection and Management	https://nrmedical.training/blog/sepsis-spot-it-early		Slurred speech or confusion	Extreme shivering or muscle pain	Passing no urine (in a day)	Severe breathlessness	Skin mottled or discolored	It feels like you’re going to die			
Search #1 - Sepsis and “First Aid”	End Sepsis [43]	What Is Sepsis?	https://www.endsepsis.org/what-is-sepsis/	Fever and chills	Confusion or sleepiness	Extreme pain		Rapid breathing	Pale or mottled skin	Feeling the sickest you've ever felt	Fast heartbeat		
Search #1 - Sepsis and “First Aid”	TLCT [44]	Could It Be Sepsis? Local First Aid Training Courses	https://www.tlct.co.uk/could-it-be-sepsis/		Slurred speech or confusion	Extreme shivering or muscle pain	Passing no urine (in a day)	Severe breathlessness	Skin mottled or discolored	It feels like you’re going to die			
Search #2 - "Sepsis Recognition" and Public	Maryland Department of Health - Sepsis Public Awareness Campaign Workgroup [45]	Sepsis Public Awareness Campaign Workgroup Meeting #1 Minutes	https://health.maryland.gov/phpa/IDEHASharedDocuments/Minutes%20for%20Sepsis%20Workgroup%20Meeting%201.cc5.pdf	Abnormal temperature	Mental decline	Extreme pain and discomfort		Shortness of breath	Discolored skin				Signs and symptoms of an infection
Search #3 - "Sepsis Recognition" and "Signs and Symptoms"	CDC (Centers for Disease Control and Prevention) [46]	Get Ahead of Sepsis	https://www.cdc.gov/sepsis/media/pdfs/Consumer-brochure-its-time-to-talk-about-sepsis-2022-P.pdf	Fever or feeling very cold	Confusion or disorientation	Extreme pain or discomfort; shivering		Shortness of breath	Clammy or sweaty skin		High heart rate or weak pulse		
Search #3 - "Sepsis Recognition" and "Signs and Symptoms"	Mayo Clinic [47]	Sepsis	https://www.mayoclinic.org/diseases-conditions/sepsis/symptoms-causes/syc-20351214		Change in mental status Feeling lightheaded	Shivering		Fast, shallow breathing	Sweating for no clear reason				Symptoms specific to the type of infection, such as painful urination from a urinary tract infection or worsening cough from pneumonia
Search #3 - "Sepsis Recognition" and "Signs and Symptoms"	Penn Medicine [48]	Sepsis	https://www.pennmedicine.org/for-patients-and-visitors/patient-information/conditions-treated-a-to-z/sepsis	Fever or low body temperature (hypothermia); Chills	Confusion or delirium; lightheadedness due to low blood pressure				Skin rash or mottled skin; warm skin		Rapid heartbeat		
Search #3 - "Sepsis Recognition" and "Signs and Symptoms"	World Health Organization [49]	Sepsis	https://www.who.int/news-room/fact-sheets/detail/sepsis	Fever or low temperature	Confusion	Extreme body pain or discomfort; shivering	Low urine output	Difficulty breathing	Clammy and sweaty skin		High heart rate, weak pulse, or low blood pressure		
Search #3 - "Sepsis Recognition" and "Signs and Symptoms"	NHS (UK) [50]	Sepsis	https://www.nhs.uk/conditions/sepsis/		Acting confused, slurred speech, or not making sense			Difficulty breathing, breathlessness or breathing very fast	Blue, gray, pale or blotchy skin, lips or tongue – on brown or black skin; this may be easier to see on the palms of the hands or soles of the feet; A rash that does not fade when you roll a glass over it, the same as meningitis				
Search #3 - "Sepsis Recognition" and "Signs and Symptoms"	Texoma Medical Center [51]	Recognizing Signs of Sepsis	https://www.texomamedicalcenter.net/recognizing-signs-sepsis	Temperature: higher or lower than normal	Mental decline: confused, sleepy, difficult to rouse	Severe pain or discomfort				Extremely Ill: "I feel like I might die"			May have signs or symptoms of an infection
Search #3 - "Sepsis Recognition" and "Signs and Symptoms"	Sepsis Alliance [52]	It's about TIME	https://www.sepsis.org/about/its-about-time/	Temperature: higher or lower than normal	Mental decline: confused, sleepy, difficult to rouse	Extremely Ill: severe pain, discomfort		Shortness of breath					May have signs and symptoms of an infection
Search #3 - "Sepsis Recognition" and "Signs and Symptoms"	AARP [53]	7 Sepsis Symptoms You Should Recognize	https://www.aarp.org/health/conditions-treatments/info-2023/sepsis-symptoms.html	Fever and chills; A very low body temperature	Lethargy or tiredness; confusion or dizziness	Extreme pain or discomfort (often at the infection site); shivering		Fast breathing or breathlessness	Clammy, sweaty or blotchy skin		Fast heart rate		
Search #3 - "Sepsis Recognition" and "Signs and Symptoms"	The UK Sepsis Trust [54]	About Sepsis	https://sepsistrust.org/about/about-sepsis/		Slurred speech or confusion	Extreme shivering or muscle pain	Passing no urine (in a day)	Severe breathlessness	Skin mottled or discolored	It feels like you’re going to die			
Search #3 - "Sepsis Recognition" and "Signs and Symptoms"	Cleveland Clinic [55]	Sepsis	https://my.clevelandclinic.org/health/diseases/12361-sepsis	Fever or hypothermia (very low body temperature)	Confusion or agitation	Low energy/weakness; shaking or chills; extreme pain or discomfort	Urinary issues, such as reduced urination or an urge to urinate	Hyperventilation (rapid breathing) or shortness of breath	Rash makes your skin appear red and discolored; You may see small, dark-red spots on your skin; warm or clammy/sweaty skin		Fast heart rate; low blood pressure		
Search #3 - "Sepsis Recognition" and "Signs and Symptoms"	National Kidney Foundation [56]	Sepsis	https://www.kidney.org/atoz/content/sepsis	Temperature: higher or lower than normal; fever, or feeling very cold	Mental decline: confused, sleepy, difficult to rouse; confusion or disorientation	Severe pain or discomfort; shivering		Extremely Ill: shortness of breath	Clammy or sweaty skin		High heart rate or weak pulse		May have signs or symptoms of an infection
Search #3 - "Sepsis Recognition" and "Signs and Symptoms"	Australian Commission on Safety and Quality in Health Care [57]	Quality Statement 1 - Could It Be Sepsis?	https://www.safetyandquality.gov.au/standards/clinical-care-standards/sepsis-clinical-care-standard/quality-statements/quality-statement-1-could-it-be-sepsis	Fever and chills; low body temperature	Fatigue, confusion, or sleepiness	A lot of pain	Low or no urine output	Fast breathing or breathlessness		‘feeling worse than ever’	Fast heartbeat	Nausea and vomiting; diarrhoea	
Search #3 - "Sepsis Recognition" and "Signs and Symptoms"	Queensland Government [58]	Adult Sepsis	https://clinicalexcellence.qld.gov.au/priority-areas/safety-and-quality/sepsis/adult-sepsis	Fever or hypothermia	New onset confusion or altered consciousness	Extreme pain; weakness or aching muscles	Poor urine output	Rapid breathing	Skin rash or clammy/sweaty skin	Feeling very unwell unexplained or the “worst ever”; feeling "the sickest" they have ever been or feeling "an impending sense of doom'"	Rapid heart rate		

Discussion

This topic was selected by the ILCOR First Aid Task Force as a significant proportion of preventable deaths are caused by sepsis worldwide, and there are known benefits of early detection and treatment. No prior review has been undertaken, and in 2022, the Task Force elected by consensus to undertake a scoping review on the recognition and awareness of sepsis by first-aid providers evaluating adults with an acute illness. There were insufficient studies identified to support a systematic review. 

Sepsis initiatives have increasingly focused on early sepsis recognition by the lay provider as a means to decrease time to hospital presentation and management with antibiotics and initial resuscitation. Despite the utilization of early warning scoring tools by trained clinicians in the healthcare setting to assist in the detection of sepsis, sepsis recognition remains a challenge due to the variable reliability of the scoring tools. Based on evidence from the EMS and in-hospital settings, no specific sign or symptom or compilation of signs and symptoms has demonstrated a clear association with sepsis. Therefore, it is unreasonable to expect a lay provider to recognize and subsequently diagnose an acute illness as sepsis using only the signs and symptoms exhibited by an ill person. A more feasible request of a lay provider is to, at a minimum, consider an infection in a person being evaluated with an acute illness.

It was noted that online resources providing education to the public on sepsis recognition listed presenting signs and symptoms of sepsis under nine general categories (Table 4): temperature (fever or hypothermia), neurologic (change in mental state, dizziness, slurred speech), musculoskeletal (severe muscle pain, extreme shivering), urologic (poor urine output), respiratory (rapid breathing or breathlessness), skin (clammy/sweaty, new rash, mottled or discolored), cardiac (elevated heart rate), gastrointestinal (nausea, vomiting, diarrhea), and subjective (feeling very unwell or impending sense of doom). However, it was variable as to which signs or symptoms were highlighted by each campaign or organization. For instance, only five of the 27 websites included from the gray literature search mentioned “infection” when describing presenting signs and symptoms of sepsis. Additionally, the presence of a fever (≥ 38°C) was noted to be inconsistently listed as a sign of sepsis and highlights that an elevated temperature is not a prerequisite for this diagnosis.

**Table 4:** Categories of Signs and Symptoms from Sepsis Awareness Campaigns

Categories	Examples
Subjective	Feeling very unwell or impending sense of doom
Temperature	Fever or hypothermia
Respiratory	Rapid breathing or breathlessness
Cardiac	Elevated heart rate
Neurologic	Change in mental state, dizziness, slurred speech
Gastrointestinal	Nausea, vomiting, diarrhea
Musculoskeletal	Severe muscle pain or extreme shivering
Urologic	Poor urine output
Skin	Clammy/sweaty, new rash, mottled or discolored

It is noteworthy that no literature originating from LMICs was included in this scoping review. Owing to a greater burden of infectious diseases and delayed presentations due to poor access to healthcare, the LMIC contexts may have much higher incidences of sepsis, resulting in higher mortality [4,5]. It is also unclear to what extent the signs and symptoms reported in the HIC literature are transferable to LMICs, where diarrheal illnesses or vector-borne diseases (such as malaria) predominate. In LMICs that feature developing or do not have emergency care systems and services, the integration of community-based first-aid providers offers a prime opportunity to increase sepsis recognition and initiate healthcare-seeking behavior [59], ultimately resulting in decreased mortality. Given a higher sepsis incidence and the critical role that a first-aid provider might play in such nascent systems, it is necessary that research in LMIC contexts should be advanced.

Limitations

There are several limitations to this review. Although this scoping review has not identified sufficient evidence to prompt a further systematic review, it highlights important gaps in research, specifically in the first-aid setting. Retrospective diagnostic studies are needed to evaluate the accuracy of criteria used in specific sepsis awareness campaigns for lay providers. The effectiveness of sepsis awareness campaigns in helping lay responders identify sepsis should be studied to determine if any one campaign is more helpful than the other. Additionally, none of the included studies were conducted in LMICs where access of the disadvantaged to sepsis care or education may have led to alternative results.

## Conclusions

Given the diagnostic challenge faced in the first-aid setting and time-sensitive nature of sepsis, increasing sensitivity for the detection of sepsis can be achieved by screening people with an infection and any correlating signs or symptoms that may fall along the spectrum of a less severe presentation. Although the criteria for sepsis may not be fulfilled, at that time or anytime in the future, they are likely to still benefit from an evaluation by a medical professional. Therefore, a first-aid provider should consider an infection in a person who presents with an acute illness, and if associated with any abnormal signs or symptoms, recommend seeking further medical evaluation.

This scoping review found no studies directly addressing our outcomes. However, we identified studies that evaluated patients in the prehospital and in-hospital settings for potential sepsis, as well as awareness and knowledge of sepsis by various members of the community. The limited literature identified in this scoping review does not support the development of a systematic review but highlights the need for future research to institute measures for the recognition of sepsis in first-aid settings.

## Appendices

Appendix 1 

**Table 5:** Initial Search Query in and Results from Embase and Medline databases (January 17, 2023)

No.	Search String Query (January 17, 2023)	Results
1	Emergency Medical Services/ or Emergency Medical Technicians/ or Emergency Services/ or First Aid/	180,403
2	("emergency care" or "emergency health service*" or "emergency medical service*" or emergicenter* or "medical emergency service*" or "prehospital emergency care" or emergicentre* or emergi-center* or emergi-centre* or "pre-hospital emergency care" or EMT* or "emergency medical technician*" or "first responder*" or "first aid" or "critical care").ti,ab,kw,kf.	279,299
3	1 or 2 [FIRST AID]	418,551
4	exp Sepsis/	459,583
5	sepsis (or sepses or septic or "bloodstream infection*" or pyemi* or pyohemi* or pyaemi* or septicemi* or septicaemi* or "blood poisoning*" or bacteremi* or fungemi* or candidemi*).ti,ab,kw,kf.	519,778
6	4 or 5 [SEPSIS]	681,253
7	3 and 6 [FIRST AID + SEPSIS]	17,089
8	(evaluat* or assess*).ti,ab,kw,kf.	16,348,275
9	7 and 8 [FIRST AID + SEPSIS + EVALUATE]	7066
10	(Animals/ or "Animal Experimentation"/ or "Models, Animal"/ or "Disease Models, Animal"/) not (Humans/ or "Human Experimentation"/)	8,804,236
11	9 not 10 [ANIMAL STUDIES REMOVED]	6865
12	(comment or editorial or "newspaper article" or news or note or lecture).pt.	3,322,316
13	(letter not (letter and randomized controlled trial)).pt.	2,452,749
14	11 not (12 or 13) [OPINION PIECES REMOVED]	6769
15	"case reports".pt.	2,313,061
16	14 not 15 [CASE REPORTS REMOVED]	6738
17	(conference or conference abstract or "conference review" or congresses).pt.	5,438,450
18	16 not 17 [CONFERENCES REMOVED]	5076
19	limit 18 to english	4726
20	limit 18 to abstracts	5055
21	19 or 20	5074
	Embase <1974 to 2023 January 17>	3238
	Ovid MEDLINE(R) and Epub Ahead of Print, In-Process, In-Data-Review & Other Non-Indexed Citations and Daily <1946 to January 17, 2023>	1836
22	remove duplicates from 21	3554
	Embase <1974 to 2023 January 17>	1722
	Ovid MEDLINE(R) and Epub Ahead of Print, In-Process, In-Data-Review & Other Non-Indexed Citations and Daily <1946 to January 17, 2023>	1832

**Table 6:** Initial Search Query in and Results from the Cochrane database (January 17, 2023)

No.	Search String Query (January 17, 2023)	Results
#1	("emergency care" OR "emergency health service" OR "emergency medical service" OR "medical emergency service" OR "emergency health services" OR "emergency medical services" OR emergicenter* OR "medical emergency service" OR "medical emergency services" OR "prehospital emergency care" OR emergicentre* OR emergi-center* OR emergi-centre* OR "pre-hospital emergency care" OR EMT* OR "emergency medical technician" OR "first responder" OR "emergency medical technicians" OR "first responders" OR "first aid" OR "critical care"):ti,ab,kw	12,546
#2	(sepsis OR sepses OR septic OR "bloodstream infection" OR "bloodstream infections" OR pyemi* OR pyohemi* OR pyaemi* OR septicemi* OR septicaemi* OR "blood poisoning" OR "blood poisonings" OR bacteremi* OR fungemi* OR candidemi*):ti,ab,kw	18,820
#3	#1 AND #2	759
#4	(evaluat* OR assess*):ti,ab,kw	961,602
#5	#3 AND #4	489
#6	([mh ^Animals] OR [mh ^"Animal Experimentation"] OR [mh ^"Models, Animal"] OR [mh ^"Disease Models, Animal"]) NOT ([mh ^Humans] OR [mh ^"Human Experimentation"])	5
#7	#5 NOT #6	489
#8	(comment OR editorial OR "newspaper article" OR news OR note OR lecture):pt	13,136
#9	(letter NOT (letter AND randomized controlled trial)):pt	13,250
#10	#7 NOT (#8 OR #9)	483
#11	"case reports":pt	0
#12	#10 NOT #11	483
#13	(conference OR "conference abstract" OR "conference review" OR congresses):pt	215,178
#14	#12 NOT #13	420
#15	#12 NOT #13 in Cochrane Reviews, Trials (15 Cochrane Reviews, 405 Trials)	420

Appendix 2

**Table 7:** Follow-Up Search Query in and Results from Embase database (November 21, 2023)

No.	Search String Query (November 21, 2023)	Results
#1	'emergency treatment'/de OR 'first aid'/de OR 'first responder (person)'/exp	39,589
#2	'emergency care':ti,ab,kw OR 'emergency health service*':ti,ab,kw OR 'emergency medical service*':ti,ab,kw OR emergicenter*:ti,ab,kw OR ' medical emergency service*':ti,ab,kw OR 'prehospital emergency care':ti,ab,kw OR emergicentre*:ti,ab,kw OR 'emergi center*':ti,ab,kw OR 'emergi centre*':ti,ab,kw OR 'pre-hospital emergency care':ti,ab,kw OR emt*:ti,ab,kw OR 'emergency medical technician*':ti,ab,kw OR 'first responder*':ti,ab,kw OR 'first aid':ti,ab,kw OR 'critical care':ti,ab,kw	181,454
#3	#1 OR #2	60,6221
#4	'sepsis'/exp OR 'bloodstream infection'/exp	388,544
#5	sepsis:ti,ab,kw OR sepses:ti,ab,kw OR septic:ti,ab,kw OR 'bloodstream infection*':ti,ab,kw OR pyemi*:ti,ab,kw OR pyohemi*:ti,ab,kw OR pyaemi*:ti,ab,kw OR septicemi*:ti,ab,kw OR septicaemi*:ti,ab,kw OR 'blood poisoning*':ti,ab,kw OR bacteremi*:ti,ab,kw OR fungemi*:ti,ab,kw OR candidemi*:ti,ab,kw	329,119
#6	#4 OR #5	484,918
#7	#3 AND #6	32,335
#8	#7 AND ('Conference Abstract'/it OR 'Conference Paper'/it OR 'Conference Review'/it OR 'Editorial'/it OR 'Letter'/it OR 'Note'/it)	12,593
#9	#7 NOT #8	19,742
#10	evaluat*:ti,ab,kw OR assess*:ti,ab,kw	10,132,567
#11	#9 AND #10	7047
#12	#11 NOT ([animals]/lim NOT [humans]/lim)	6661
#13	#11 NOT ([animals]/lim NOT [humans]/lim) AND [english]/lim	6265
#14	#11 NOT ([animals]/lim NOT [humans]/lim) AND [english]/lim AND [2023-current]/py	228

**Table 8:** Follow-Up Search Query in and Results from Medline database (November 21, 2023)

No.	Search String Query (November 21, 2023)	Results
1	Emergency Medical Services/	49,123
2	Emergency Medical Technicians/	6060
3	First Aid/	8144
4	1 or 2 or 3	59,817
5	("emergency care" or "emergency health service*" or "emergency medical service*" or emergicenter* or "medical emergency service*" or "prehospital emergency care" or emergicentre* or emergi-center* or emergi-centre* or "pre-hospital emergency care" or EMT* or "emergency medical technician*" or "first responder*" or "first aid" or "critical care").ti,ab,kw,kf	120,880
6	4 or 5	163,765
7	Exp Sepsis/	143,368
8	(sepsis or sepses or septic or "bloodstream infection*" or pyemi* or pyohemi* or pyaemi* or septicemi* or septicaemi* or "blood poisoning*" or bacteremi* or fungemi* or candidemi*).ti,ab,kw,kf.	222,148
9	7 or 8	273,624
10	6 and 9	5229
11	(evaluat* or assess*).ti,ab,kw,kf.	7,258,332
12	10 and 11	2125
13	(Animals/ or "Animal Experimentation"/ or "Models, Animal"/ or "Disease Models, Animal"/) not (Humans/ or "Human Experimentation"/)	5,140,014
14	12 not 13	2081
15	(comment or editorial or "newspaper article" or news or note or lecture).pt.	1,719,034
16	(letter not (letter and randomized controlled trial)).pt.	1,230,350
17	14 not (15 or 16)	2038
18	"case reports".pt.	2,369,855
19	17 not 18	2006
20	(conference or conference abstract or "conference review" or congresses).pt.	0
21	19 not 20	2006
22	limit 21 to english language	1855
23	limit 22 to yr="2023 -Current"	182

Appendix 3

Cochrane Follow-Up Search String (November 21, 2023)

("emergency care" OR "emergency health service" OR "emergency medical service" OR "medical emergency service" OR "emergency health services" OR "emergency medical services" OR emergicenter* OR "medical emergency service" OR "medical emergency services" OR "prehospital emergency care" OR emergicentre* OR emergi-center* OR emergi-centre* OR "pre-hospital emergency care" OR EMT* OR "emergency medical technician" OR "first responder" OR "emergency medical technicians" OR "first responders" OR "first aid" OR "critical care") in Title Abstract Keyword AND (sepsis OR sepses OR septic OR "bloodstream infection" OR "bloodstream infections" OR pyemi* OR pyohemi* OR pyaemi* OR septicemi* OR septicaemi* OR "blood poisoning" OR "blood poisonings" OR bacteremi* OR fungemi* OR candidemi*) in Title Abstract Keyword AND (evaluat* OR assess*) in Title Abstract Keyword - (Word variations have been searched).

Appendix 4

**Table 9:** Literature Search Query in and Results from Google.com (August 29, 2023)

#	Google.com Search	Results	Results Screened	Total Records Reviewed	Duplicate	Excluded	Included After Review
1	Sepsis and “First Aid”	747,000	100	19	2	4	13
2	"Sepsis Recognition" and Public	15,900	100	6	3	2	1
3	“Sepsis Recognition” and “Signs and Symptoms”	38,300,000	100	14	0	1	13
							Total: 27
